# Acute Intrathoracic Migration With Incarceration of Laparoscopic Sleeve Gastrectomy Due to Incomplete Hiatal Hernia Repair

**DOI:** 10.7759/cureus.31362

**Published:** 2022-11-11

**Authors:** Muhammed Said Dalkılıç, Hasan Erdem, Mehmet Gençtürk, Merih Yılmaz, Abdullah Sisik

**Affiliations:** 1 Department of General Surgery, Marmara University, Istanbul, TUR; 2 Department of General Surgery, Dr. Hasan Erdem (HE) Obesity Clinic, Istanbul, TUR

**Keywords:** incarceration, bariatric surgery complications, gastroesophageal reflux, sleeve gastrectomy, hiatal hernia

## Abstract

Concomitant hiatal hernia repair during laparoscopic sleeve gastrectomy (LSG) is recommended if it is detected. Intrathoracic sleeve migration (ITSM) is a sliding hiatal hernia that develops after LSG. In this article, we present an early ITSM due to an incomplete repair of a hiatal hernia.

An obese patient had hiatal hernia in the preoperative endoscopy. After LSG, the defect was repaired with anterior cruroplasty. Vomiting attacks were observed after the operation. Based on clinical signs and radiological findings, laparoscopic exploration was indicated. During the reoperation, an acute entrapment of the upper portion of the sleeve was observed, which had migrated through the hiatus. This suture was undone. There was no gastric ischemia. No additional hiatal repair was attempted.

The operation was sufficient to alleviate the symptoms. The patient was discharged on the second postoperative day uneventfully. Until the most recent follow-up, the patient has progressed with adequate weight loss, without complaints of reflux and without proton pump inhibitors

ITSM with incarceration is a complication that can occur after incomplete hiatal repair. Failure to perform hiatal repair with proper technique can be attributed to this complication.

## Introduction

Laparoscopic sleeve gastrectomy (LSG), due to its technical ease and low morbidity and complication rates, has become the most commonly used method in the surgical treatment of morbid obesity [1]. In addition to well-known complications such as leakage and bleeding, intrathoracic sleeve migration (ITSM) and acute incarcerated hiatal hernia have also been described and are seen less frequently. ITSM can be defined as a sliding hiatal hernia that develops after LSG, and there is a possibility of incarceration [2].

The majority of patients who are scheduled for LSG for the treatment of obesity have hiatal hernia, and it is thought to play an important role in gastroesophageal reflux disease (GERD), which occurs at a rate of 25-75% after surgery [3]. The results of concomitant hiatal hernia repair, which is one of the suggested strategies to reduce GERD after LSG, are controversial [4]. Conservative and surgical repair approaches have been described in the literature for concomitant hiatal hernias detected during LSG.

In this study, we present a case of an acute incarcerated hiatal hernia due to intrathoracic sleeve migration (ITSM) in a patient who underwent concomitant hiatal repair with LSG.

## Case presentation

A 36-year-old female patient (BMI 40 kg/m2) without any disease or surgical history was admitted to our center for the surgical treatment of obesity. Hill Grade 4 hiatal hernia was detected in the endoscopy performed routinely during the preoperative evaluation. It was decided to perform LSG in the patient who was asymptomatic in terms of reflux. Informed consent was obtained from the patient for possible hiatal hernia repair.

LSG was performed with our standard technique: After entering the abdomen with four trocars, the gastrocolic omentum was dissected along the greater curvature, starting 4 cm from the pylorus. The left crus and phrenoesophageal ligament were dissected until the fundus was completely freed. At this time, a very large hiatal defect was observed. After the 36 Fr orogastric tube was placed, vertical sleeve gastrectomy was performed with an endovascular gastrointestinal anastomosis (Endo-GIA) stapler, starting 4 cm from the pylorus. Then, the right and left crus were dissected and a large hiatal defect was revealed. The anterior repair was performed with a 2/0 prolene suture without removing the orogastric tube. After the repair, no occlusion was observed.

The patient described difficulty in swallowing against water in the postoperative period. Vomiting attacks that did not respond to medical treatment were observed. It was observed that the attacks continued in clear liquid intake on the second postoperative day. Based on clinical signs and radiological findings, laparoscopic exploration was promptly indicated. During the reoperation, an acute entrapment of the upper portion of the sleeve was observed, which had migrated through the esophageal hiatus. The previous stitch was in place, but loose. This suture was undone and the gastric tube was pulled into the abdomen. There were no signs of gastric ischemia. No additional esophageal hiatus repair was attempted in the reoperation. The gastrocolic ligament was then sutured to the sleeve staple line in an attempt to steady the gastric tube within the abdominal cavity, reducing the possibility of additional intrathoracic migration. The hiatal defect was left open. These two operations and the contrast X-ray are shown in Video 1. 

**Video 1:** Acute intrathoracic migration with incarceration of laparoscopic sleeve gastrectomy with incomplete hiatal hernia repair

Written consent was obtained from the patient for the publication of this case report and accompanying images. Since this publication is a case report, the Institutional Review Board was not required or needed.

Opening the esophageal hiatus by removing the previous stitch was sufficient to alleviate the symptoms resulting from the acute incarceration, and the patient was discharged on the second postoperative day, uneventfully. Until the most recent follow-up (sixth postoperative month), the patient has progressed with adequate weight loss (29% of total weight loss), without significant complaints of gastroesophageal reflux and without regular use of proton pump inhibitors.

## Discussion

A clinical case involving an early post-sleeve complication, most likely due to a defective concomitant hiatal hernia repair, is presented in this article. 

ITSM can be a major cause of nausea, dysphagia, and postoperative reflux disease. Because of the risk of up to 50% in the long term after surgery, it is mostly discussed in the context of LSG [5].

Failure to recognize and repair a hiatal hernia at the time of initial operation may lead to reflux and intrathoracic migration of the upper sleeve. In hiatal hernia repair, the distal esophagus must be extensively mobilized and pulled into the abdominal cavity, where it must be positioned stably. In this case, the absence of preoperative reflux symptoms led us to opt for a simpler and faster hiatal repair technique, which due to incomplete dissection of the distal esophagus, may have favored the ITSM. 

The results of concomitant hiatal repair with LSG are controversial. Although there are studies claiming that the repair is ineffective, the general recommendation is to repair the hiatal hernia. In the SAGES Manual of Bariatric Surgery published in 2018, it was stated that if there is a hiatal defect, it should be repaired with non-absorbable sutures in the same surgery [6]. Remarkably, 82% of the panelists at the International Sleeve Gastrectomy Expert Panel Consensus in 2011 revealed that they believed concomitant hiatal hernia should be repaired [7]. In the study of Boru et al. in which they evaluated the success of hiatal hernia repair performed to prevent postoperative reflux and gave their results after a five-year follow-up, the success rates were found to be 82% (48 patients) after suture hiatoplasty and 96% after hiatoplasty reinforced with bioabsorbable mesh (48 patients) [8].

Samakar et al. reported that symptoms did not improve in 76% of 26 symptomatic patients with concomitant hiatal hernia repair during LSG, and new-onset GERD was detected in 16% of 32 previously asymptomatic patients. In the study of Santanicola et al., after a minimum follow-up of six months, the prevalence of GERD in the LSG + hiatal repair group did not show a significant change (from 38.4% to 30.8%) but showed a significant decrease in the LSG-only group (from 39.2% to 19.6%, or p= 0.003). The authors even emphasized that concomitant hiatal hernia repair may be a promotor factor for GERD [9,10].

The pathophysiology of sleeve incarceration after concomitant hiatal hernia repair; It is possible to explain it with dissection of the phrenoesophageal membrane to free the fundus and recurrent, uncontrolled retching and vomiting in the early postoperative period [11]. When the esophagus and stomach become mobile after the crus and hiatus dissection, the risk of reflux, transthoracic migration, and twist increases [3]. Crurography for defect repair may also increase the risk of incarceration. It can be thought that the factor preventing migration in crus repair performed with fundoplication is the prepared fundoplication. In the crus repair performed together with LSG, there is no factor to prevent migration; on the contrary, structures such as the phrenoesophageal ligament and greater omentum that provide stabilization are dissected. In our opinion, one of the precautions to be taken here is to ensure the stability of the stomach, which has become mobile and prone to migration. This can be done by several methods: During the crus repair, the suture can be passed through the stomach or abdominal esophagus [11], or omentopexy can be performed as in our patient. The omentopexy choice for preventing re-migration is debatable and not based on literature. Another precaution is to add an anti-reflux procedure to the LSG, such as the Hill procedure [12]. Hiatoplasty or cardiopexy with the help of ligamentum teres hepatis is a technique that has been recently suggested [6,13]. Mesh reinforcement with hiatoplasty can be performed in selected patients [14]. Although all these techniques are interesting, their long-term results are still unknown and their routine use removes the most attractive feature of LSG, its technical simplicity [5]. 

To the best of our knowledge, ITSM with acute incarceration in the early postoperative period due to incomplete concomitant hiatal hernia repair has not been reported before. 

## Conclusions

ITSM with acute incarceration is a complication that can occur after mistakenly concomitant hiatal hernia repair. This emergency should always be suspected in post-sleeve patients with concomitant hiatal hernia repair who have persistent nausea and vomiting. Failure to perform hiatal dissection and repair with proper technique can be attributed to this complication.
